# Efficacy of targeted therapy for advanced renal cell carcinoma: A systematic review and meta-analysis of randomized controlled trials

**DOI:** 10.1590/S1677-5538.IBJU.2017.0315

**Published:** 2018

**Authors:** Chao Wei, Shen Wang, Zhangqun Ye, Zhiqiang Chen

**Affiliations:** 1 Department of Urology, Tongji Hospital, Tongji Medical College, Huazhong University of Science and Technology, Wuhan, China; 2 Institute of Urology, Tongji Hospital, Tongji Medical College, Huazhong University of Science and Technology, Wuhan, China

**Keywords:** Carcinoma, Renal Cell, Therapeutics, Meta-Analysis as Topic, Interferons

## Abstract

We conducted a systematic review and meta-analysis of the literature on the efficacy of the targeted therapies in the treatment of advanced RCC and, via an indirect comparison, to provide an optimal treatment among these agents. A systematic search of Medline, Scopus, Cochrane Library and Clinical Trials unpublished was performed up to Jan 1, 2015 to identify eligible randomized trials. Outcomes of interest assessing a targeted agent included progression free survival (PFS), overall survival (OS) and objective response rate (ORR). Thirty eligible randomized controlled studies, total twentyfourth trails (5110 cases and 4626 controls) were identified. Compared with placebo and IFN-α, single vascular epithelial growth factor (receptor) tyrosine kinase inhibitor and mammalian target of rapamycin agent (VEGF(r)-TKI & mTOR inhibitor) were associated with improved PFS, improved OS and higher ORR, respectively. Comparing sorafenib combination vs sorafenib, there was no significant difference with regard to PFS and OS, but with a higher ORR. Comparing single or combination VEGF(r)-TKI & mTOR inhibitor vs BEV + IFN-α, there was no significant difference with regard to PFS, OS, or ORR. Our network ITC meta-analysis also indicated a superior PFS of axitinib and everolimus compared to sorafenib. Our data suggest that targeted therapy with VEGF(r)-TKI & mTOR inhibitor is associated with superior efficacy for treating advanced RCC with improved PFS, OS and higher ORR compared to placebo and IFN-α. In summary, here we give a comprehensive overview of current targeted therapies of advanced RCC that may provide evidence for the adequate targeted therapy selecting.

## INTRODUCTION

Renal cell carcinoma (RCC) accounts for about 85% of kidney cancers (1), and approximately 25-30% of patients present with advanced RCC, which is defined as metastatic and/or unresectable disease (2). Metastatic renal-cell carcinoma (mRCC) has always been one of the most drug-resistant malignancies (3) and the 5-year survival rates remain low at only around 10% and had not improved by 2008 on the basis of the National Cancer Data Base (NCDB) (4). Over the past two decades, immunomodulating drugs such as interferon-α (IFN) have been the standard first-line mRCC treatment (5), and have been considered the standard comparator in clinical trials (6). Recent advances through a better understanding of the molecular mechanisms involved in the pathogenesis of RCC have resulted in the development of drugs that target angiogenesis by either directly inhibiting vascular endothelial growth factor (VEGF)-mediated signalling or indirectly by inhibiting the mammalian target of rapamycin downstream (7). Compared with previously available treatment options, novel targeted therapies are now providing effective and manageable treatment for patients with advanced RCC with better tolerability (8). However, these targeted therapies are currently competing to be the primary choice for the first-line therapy of mRCC patients presenting a good or intermediate prognosis. As a consequence of the paucity of head-to-head data with other treatments, it is not possible to directly compare the efficacy of the targeted agents. Hence, in the absence of direct head-to-head comparison, there is a need for appropriate meta-analysis and valid indirect comparison assessment (9). As the optimal treatment algorithm for the management of advanced RCC remains to be determined, the aim of the current systematic review and meta-analysis was to demonstrate the clinical efficacy of different targeted treatments for the management of patients with advanced RCC and use indirect comparisons to provide an optimal option among these agents.

## MATERIALS AND METHODS

### Literature search and study selection

A systematic search of the electronic databases, including Medline, Scopus and Cochrane Library was performed to identify trials on the targeted therapies of advanced RCC up to January 1, 2015 which was when the search was completed. The strategy consisted of searching for publications using key terms related to the target drugs (e.g., agents’ names) and various terms used to describe renal cancer included renal cancer or renal tumor or renal neoplasm or renal carcinoma. We also sought unpublished studies through “clinicaltrials.gov”. No temporal, regional, publication status or language restrictions were set. In addition, a full manual search of the references in each relevant article was also conducted.

### Inclusion criteria and exclusion criteria

We included any randomized clinical trial evaluating the therapeutic efficacy of VEGF(r)-TKI bevacizumab, sorafenib, sunitinib, pazopanib, tivozanib, or cediranib and the mTOR inhibitor temsirolimus, everolimus for the treatment of mRCC. Studies had to evaluate one of the study drugs combination or monotherapy with a control intervention. We included trials involving patients of any age, sex, or mRCC stage. We excluded pharmacokinetic studies, nonrandomized evaluations, animal studies and laboratory studies.

### Data extraction and outcomes of interest

Two reviewers (H.B.X. and P.J.L.) extracted independently the following data including: first author, year of publication, trial name, trial phase, published journal, prior therapy, the intervention and comparator agents, number of patients and outcomes of interest. All disagreements about eligibility were resolved by a third reviewer (H.X.) by discussion until a consensus was reached. Our primary outcome was PFS, the most consistently reported endpoint. Key secondary effectiveness outcomes included OS, ORR by RECIST (Response Evaluation Criteria in Solid Tumors) criteria, and patient-reported outcomes.

### Study quality and level of evidence

The quality appraisal of included studies was analyzed using the Jadad scale (10). Two reviewers (H.B.X. and ZhL.X.) independently assessed the quality of the studies and disagreement was resolved by consensus.

### HR pooled

Hazard ratios (HRs) and 95% confidence intervals (CIs) were used to estimate the impact of targeted therapies on PFS and OS. A combined HR >1 implied a worse survival, and it was considered statistically significant if 95% CI for the combined HR did not overlap 1. For the studies in which HR was not given directly, the published data and figures from original papers were used to calculate the HR according to the methods described by Parmar et al (11). The O-E and variance were calculated from the reported data directly by HR and its 95% CI or indirectly by log-rank *P* value with number of events, or data reading from Kaplan-Meier survival curve. All *P* values are two-tailed with a significant level at 0.05. Kaplan-Meier curves were read by Engauge Digitizer version 4.1 (http://digitizer.sourceforge.net/) (12). This work was performed by two independent persons to reduce inaccuracy in the extracted survival rates. Discrepancies in these articles were resolved by discussion.

### Indirect treatment comparison

Standard indirect comparison methods were applied to independent review PFS data of the randomized trials, to indirect treatment comparison (ITC) HR with 95% CI. If there are two agents and both have been compared to another, indirect comparison was enabled by the common comparator arms. The ITC of PFS outcomes uses the most widely applied indirect comparison method by Bucher et al. (13). The PFS HRs of eligible RCTs were selected as the preferred outcome for the ITC, as this effect measure accounts for censoring and incorporates time to event information (Table-1) (14). Each trial PFS outcomes which present the highest quality data based on independent central review assessment were also selected as the basis of the ITC (Table-2). For example, trial TARGET and NCT00079612 reported the comparison between Sorafenib and placebo, while trial RECORD-1 reported Everolimus vs placebo, as Everolimus and Sorafenib have been compared to placebo, ITC was enabled by the common placebo control arms. As shown in Supplementary protocol designs (Appendix), for the trails such as TARGET and RECORD-1 in which patient characteristics, enrolment criterion, and study measurements are comparable, but not identical, ITC was conducted and the other ITCs were also carried according to this protocol. All calculations have been performed by our advanced setting program in Excel 2007 (Microsoft Office). The ITC calculations can also be reperformed using the ITC tool available from Wells et al. which ensures maximum transparency (15). We did not perform an indirect comparison of the effect of interventions on OS data because there was a lack of final OS data reported in the studies analyzed and because of uncertainty regarding post study medication usage.

**Table 1:** Pooled outcomes of included randomized trials.

Trail	PFS, months (Int vs. Con)	HR (95% CI)	P	ORR %	P	OS, months (Int vs. Con)	HR (95% CI)	P
**VEGF(r)-TKI & mTOR inhibitor vs placebo**								
NCT00019539 (16)	4.8/2.5	0.43 (0.26-0.72)	<0.0001	10 vs 0	ns	nr	0.84(0.58-1.22)	ns
NCT00079612 (17)	5.5/1.4	0.42 (0.20-0.91)	0.009	nr	nr	nr	nr	nr
TARGET (18, 19)	5.5/2.8	0.51 (0.43-0.60)	<0.0001	2 vs 0	ns	17.8/15.2	0.88(0.74-1.04)	0.146
VEG 105192 (20-22)	9.2 (7.4-12.9)/4.2 (2.8-4.2)	0.46 (0.34-0.62)	<0.0001	30 vs 3	<0.0001	22.9 (19.9-25.4)/20.5 (15.6-27.6)	0.91 (0.71-1.16)	0.224
NCT00502307 (23)	10.3 (8.1-21.2)/3.3 (1.8-8.0)	0.55 (0.33-0.91)	0.01	nr	nr,	nr	nr	nr
NCT00423332 (24)	12.1/2.76	0.45 (0.26-0.78)	0.017	34 vs 5.56	nr	nr	nr	nr
RECORD-1 (25)	4.0 (3.7-5.5)/1.9 (1.8-1.9)	0.30 (0.22-0.40)	<0.0001	1 vs 0	ns	NA/8.8 (7.9-NA)	0.83(0.50-1.37)	0.23
**VEGF(r)-TKI & mTOR inhibitor vs IFN-**α								
CALGB 90206 (26)	8.5 (7.5-9.7)/5.2 (3.1-5.6)	0.71 (0.61-0.83)	<0.0001	25.5 vs 13.1	<0.0001	nr	nr	nr
AVOREN (27-28)	5.5/2.8	0.51 (0.43-0.60)	<0.0001	31 vs 13	<0.0001	17.8/15.2	0.88(0.74-1.04)	0.146
NCT00117637 (29)	5.7 (5.0-7.4)/5.6 (3.7-7.4)	0.88 (0.61-1.27)	0.504	5.2 vs 8.6	ns	nr	nr	nr
NCT00083889 (30)	11 (10-12)/5 (4-6)	0.42 (0.32-0.54)	<0.0001	11 vs 5	0.54	114.6 (100.1-142.9)/94.9 (77.7-117)	0.65(0.45-0.94)	0.02
Global ARCC (31)	3.8 (3.6-5.2)/1.9 (1.9-2.2)	0.76 (0.62-0.92)	<0.0001	8.6 vs 4.8	ns	10.9 (8.6-12.7)/7.3 (6.1-8.8)	0.73(0.58-0.92)	0.008
**VEGF(r)-TKI & mTOR inhibitor combination vs monotherapy**								
NCT00126594 (32)	7.56 (5.19-11.07)/7.39 (5.5-9.2)	0.85 (0.51-1.42)	0.53	30 vs 25	ns	27.04 (22.31- NA)/NA	1.95(0.84-4.52)	0.122
ROSORC (33)	33/20	0.75 (0.34-1.65)	0.11	27.3 vs 14.5	ns	nr	nr	nr
NCT00467025 (34)	9.0 (5.6-13.1)/9.0 (5.5-10.9)	0.8 (0.5-1.28)	0.35	38 vs 25	ns	nr	nr	nr
Global ARCC (31)	3.7 (2.9-4.4)/3.8 (3.6-5.2)	1.08 (0.89-1.3)	ns	8.1 vs 8.6	ns	8.4 (6.6-10.3)/10.9 (8.6-12.7)	1.19(0.94-1.50)	ns
**Single VEGF(r)-TKI & mTOR inhibitor comparison**								
AXIS (35)	12.1 (8.6-NA)/4.9 (2.8-6.6)	0.39 (0.13-1.17)	0.04	52 vs 3.4	0.0001	nr	nr	nr
AXIS (36, 37)	8.3 (6.7-9.2)/5.7 (4.7-6.5)	0.66 (0.55-0.78)	<0.0001	19.4 vs 9.4	0.0001	20.1 (16.7-23.4)/19.2 (17.5-22.3)	0.97(0.80-1.17)	0.37
INTORSECT (38)	4.28 (4.01-5.43)/3.91 (2.80-4.21)	0.87 (0.71-1.07)	0.19	7.7 vs 7.9	ns	(17.5-22.3) 12.27 (10.13-14.8)/16.64(13.55-18.72)	1.31(1.05-1.63)	0.014
COMPARZ (39)	8.4 (8.3-10.9)/9.5 (8.3-11.1)	1.05 (0.90-1.22)	ns	30.7 vs 24.8		28.4 (26.2-35.6)/29.3 (25.3-32.5)	nr	nr
NCT01147822 (40)	8.4 (8.3-11.1)/11.1 (8.2-14.3)	1.02 (0.77-1.35)	ns	35.6 vs 20.7		NA (23.7-NA)/31.5(29.5-NA)	nr	nr
**VEGF(r)-TKI & mTOR inhibitor combined treatment**								
AVOREN (27)	23.3/26	0.92 (0.69-1.23)	ns	nr	nr	nr	nr	nr
RAPSODY (42)	7.9 (5.1-10.9)/8.6 (2.2-15.1)	1.35 (1.01-1.59)	0.049	17.6 vs 34	0.058	20.3 (20.5-32.4)/19.4 (23.4-36.8)	1.17(0.69-2.00)	0.412
Bukowski, 2007 (43)	9.9/8.5	0.86 (0.50-1.49)	0.58	14 vs 13	0.99	20/NA	1.57(0.84-2.94)	0.16
INTORACT (44)	9.1 (8.1-10.2)/9.3 (9.0-11.2)	1.1 (0.9-1.3)	ns	27 vs 27.4	1.0	25.8 (21.1-30.7)/25.5 (20.4-30.8)	1.0(0.9-1.3)	0.6
TORAVA (45)	8.2 (7.0-9.6)/16.8 (6.0-26.0)	1.21 (0.7-2.09)	ns	27 vs 43	nr	nr	nr	nr
TORAVA (45)	8.2 (5.5-11.7)/16.8 (6.0-26.0)	1.62 (0.84-3.16)	ns	29 vs 43	nr	nr	nr	nr

**VEGF(r)-TKI =** vascular epithelial growth factor (receptor) tyrosine kinase inhibitor; **mTOR =** mammalian target of rapamycin; **PFS =** progression free survival; **OS =** overall survival; **ORR =** objective response rate; **HR =** hazard ratio; 95% CI, 95% confidence interval; **ns =** not statistically significant; **nr =** not reported; **NA =** not attained. Data of HR estimated through Kaplan-Meier curves is indicated in italic, and remaining data is as reported by investigators.

**Table 2:** Summary of included randomized studies.

Reference	Trial name	Phase	Journal	Prior therapy	Intervention	Comparator	Patients	Outcomes
**VEGF(r)-TKI & mTOR inhibitor vs placebo**								
Yang, 2003 (16)	NCT00019539	Phase II	N Engl J Med	IL2	BEV 10 mg	Placebo	39/40	OS, PFS, ORR
Ratain, 2006 (17)	NCT00079612	Phase II	J Clin Oncol	cytokine	Sorafenib	Placebo	32/33	PFS, ORR
Escudier, 2007 (18)	TARGET	Phase III	N Engl J Med	cytokine	Sorafenib	Placebo	451/452	OS, PFS, ORR
Escudier, 2009 (19)	TARGET	Phase III	J Clin Oncol	cytokine	Sorafenib	Placebo	451/452	OS, PFS, ORR
Nieto, 2011 (20)	VEG 105192	Phase III	Clin Cancer Res	Nil, ifn α	Pazopanib	Placebo	155/78	OS, PFS, ORR
Sternberg, 2010 (21)	VEG 105192	Phase III	J Clin Oncol	Nil, ifn α	Pazopanib	Placebo	290/145	PFS, ORR
Sternberg, 2013 (22)	VEG 105192	Phase III	Eur J Cancer	Nil, ifn α	Pazopanib	Placebo	290/145	OS, ORR
Nosov, 2012 (23)	NCT00502307	Phase II	J Clin Oncol	Nil, ifn α	Tivozanib 3/1	Placebo	51/51	PFS, ORR
Mulders, 2012 (24)	NCT00423332	Phase II	Eur J Cancer	Nil	Cediranib	Placebo	53/18	PFS, ORR
Motzer, 2008 (25)	RECORD-1	Phase III	Lancet	VEGFr-TKI	Everolimus	Placebo	272/138	OS, PFS, ORR
**VEGF(r)-TKI & mTOR inhibitor vs IFN**-**α**								
Rini, 2008 (26)	CALGB 90206	Phase III	J Clin Oncol	Nil	BEV + IFN α	IFN α	369/363	PFS, ORR
Melichar, 2012 (27)	AVOREN	Phase III	ERA Ther	Nil	BEV + IFN α	IFN α	327/322	OS, PFS, ORR
Escudier, 2007 (28)	AVOREN	Phase III	Lancet	Nil	BEV + IFN α	IFN α	327/322	OS, PFS, ORR
Escudier, 2010 (29)	AVOREN	Phase III	J Clin Oncol	Nil	BEV + IFN α	IFN α	327/322	OS, ORR
Escudier, 2009 (30)	NCT00117637	Phase II	J Clin Oncol	Nil	Sorafenib	IFN α	97/92	PFS
Motzer, 2007 (31)	NCT00083889	Phase III	N Engl J Med	Nil	Sunitinib 4/2	IFN α	375/375	OS, PFS, ORR
Hudes, 2007 (32)	Global ARCC	Phase III	N Engl J Med	Nil	Temsirolimus	IFN α	209/207	OS, PFS, ORR
**VEGF(r)-TKI & mTOR inhibitor combination vs monotherapy**								
Jonasch, 2010 (33)	NCT00126594	Phase II	Cancer	Nil	Sorafenib + IFN α	Sorafenib	40/40	OS, PFS, ORR
Procopio, 2011 (34)	ROSORC	Phase II	Brit J Cancer	Nil	Sorafenib + IL2	Sorafenib	66/62	PFS, ORR
Rini, 2012 (35)	NCT00467025	Phase II	Cancer	Nil	Sorafenib + AMG386	Sorafenib	50/51	PFS, ORR
Hudes, 2007 (32)	Global ARCC	Phase III	N Engl J Med	Nil	Temsirolimus + IFN	Temsirolimus	210/209	OS, PFS, ORR
**Single VEGF(r)-TKI & mTOR inhibitor comparison**								
Ueda, 2013 (36)	AXIS	Phase III	Jpn J Clin Oncol	Any one	Axitinib	Sorafenib	25/29	PFS, ORR
Motzer, 2013 (37)	NCT00678392	Phase III	Lancet Oncol	Any one	Axitinib	Sorafenib	361/362	PFS, OS, ORR
Rini, 2011 (38)	AXIS	Phase III	Lancet	Any one	Axitinib	Sorafenib	361/362	PFS, ORR
NCT00474786 (39)	INTORSECT	Phase III	unpublished	Sunitinib	Temsirolimus	Sorafenib	259/253	OS, PFS, ORR
Celler, 2013 (40)	COMPARZ	Phase III	J Clin Oncol	Nil	Pazopanib	Sorafenib	557/553	OS, PFS, ORR
NCT01147822 (41)	NCT01147822	Phase II	unpublished	Nil	Pazopanib	Sunitinib	188/179	OS, PFS, ORR
**VEGF(r)-TKI & mTOR inhibitor combined treatment**								
Escudier, 2010 (29)	AVOREN	Phase III	J Clin Oncol	Nil	BEV + LD-IFN α	BEV + IFN α	13/3271	OS, ORR
Bracarda, 2007 (42)	RAPSODY	Phase II	Eur Urol	Nil	Sorafenib + IFN α×5	Sorafenib + IFN α	51/50	OS, PFS, ORR
Bukowski, 2007 (43)	NCT00081614	Phase II	J Clin Oncol	Nil	BEV + Erlotinib	BEV + Placebo	53/51	PFS, OS, ORR
NCT00631371 (44)	INTORACT	Phase III	unpublished	Nil	BEV + Everolimus	BEV + IFN α	400/391	PFS, OS, ORR
Négrier, 2011 (45)	TORAVA	Phase II	Lancet Oncol	Nil	BEV + Temsirolimus	BEV + IFN α	88/41	PFS, ORR
Négrier, 2011 (45)	TORAVA	Phase II	Lancet Oncol	Nil	Sunitinib	BEV + IFN α	42/41	PFS, ORR

**VEGF(r)-TKI =** vascular epithelial growth factor (receptor) tyrosine kinase inhibitor; **mTOR =** mammalian target of rapamycin; **PFS =** progression free survival; **OS =** overall survival; **ORR =** objective response rate; **IFN-α** = interferon-α; **BEV =** bevacizumab. Primary outcome in each study is indicated in bold.

#### Statistical analysis

We performed the meta-analysis by using the Review Manager Software (RevMan 5.1, Cochrane Collaboration, Oxford, UK). χ^2^ and I^2^ statistics were used directly to examine the heterogeneity between each study. By heterogeneity test, if I^2^<50%, we select the fixed-effect model, and if not, a random-effect model was used. We used HR, risk ratio (RR) and their CIs to evaluate the relationship between the targeted therapies and survival and ORR in advanced RCC, respectively. To test the publication bias, we used the RevMan 5.1 statistical software to make the funnel plot. P<0.05 was considered as significant difference.

## RESULTS

### Characteristics of included studies and study quality

**Figure 1:**
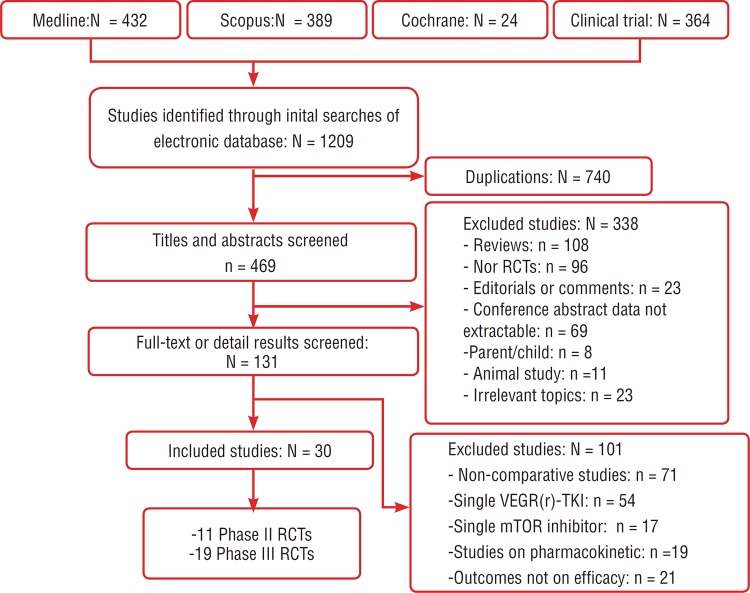
Flow diagram showing the selection process of included studies.

Thirty eligible randomized controlled studies, total twenty-four trails (5110 cases and 4626 controls) were identified (Figure-1). 19 studies were Phase III, international, multicenter, randomized clinical trials; and 11 studies were Phase II trials. There were 10 placebo control RCTs and 7 control RCTs. 4 studies compared combination vs monotherapy, 6 studies conducted comparison between single VEGF(r)-TKI & mTOR inhibitor and 5 studies performed comparison between combined treatments. First author, year of publication, trial name, trial phase, published journal, prior therapy, the intervention and comparator agents, number of patients and outcomes of interest were extracted individually from each study and listed on Table-2. We utilized the Jadad scale to assess the quality of every study included in our meta-analysis. Above the 30 RCTs (16-45) twenty-four studies (16-23, 25, 26, 28, 45) scored a 5 because the description of randomization and technique was adequate. By contrast, the other six studies (24, 27, 30, 33, 35, 36) scored a 3 on the Jadad scale because the description of double-blind or the method of blinding was inappropriate (Supplementary Table-1). In addition, according to Jadad scale and Oxford Centre for Evidence--based Medicine Levels of Evidence, we judged the strength of evidence of every study included in our meta-analysis to be Ib. Also, the effectiveness outcomes including PFS, OS, ORR and pooled HR were extracted in Table-2.

**Supplementary Table 1:** Quality assessments for each study with Jadad scale.

Questions	Randomized Study?	Randomization technique described and adequate	Randomization technique described and inadequate	Double blinded study?	Technique of blinding described and adequate	Technique of blinding described and inadequate	Description of withdrawals and dropouts?	Jaded score
Answer	Yes/No	Yes	Yes	Yes/No	Yes	Yes	Yes/No	
Score	+1/0	+1	-1	+1/0	+1	-1	+1/0	
Yang, 2003 [1]	Yes	Yes	No	Yes	Yes	No	Yes	5
Ratain, 2006 [2]	Yes	Yes	No	Yes	Yes	No	Yes	5
Escudier, 2007 [3]	Yes	Yes	No	Yes	Yes	No	Yes	5
Escudier, 2009 [4]	Yes	Yes	No	Yes	Yes	No	Yes	5
Nieto, 2011 [5]	Yes	Yes	No	Yes	Yes	No	Yes	5
Sternberg, 2010 [6]	Yes	Yes	No	Yes	Yes	No	Yes	5
Sternberg, 2013 [7]	Yes	Yes	No	Yes	Yes	No	Yes	5
Nosov, 2012 [8]	Yes	Yes	No	Yes	Yes	No	Yes	5
Mulders, 2012 [9]	Yes	Yes	No	No	No	No	Yes	3
Motzer, 2008 [10]	Yes	Yes	No	Yes	Yes	No	Yes	5
Rini, 2008 [11]	Yes	Yes	No	Yes	Yes	No	Yes	5
Melichar, 2012 [12]	Yes	Yes	No	No	No	No	Yes	3
Escudier, 2007 [13]	Yes	Yes	No	Yes	Yes	No	Yes	5
Escudier, 2010 [14]	Yes	Yes	No	Yes	Yes	No	Yes	5
Escudier, 2009 [15]	Yes	Yes	No	No	No	No	Yes	3
Motzer, 2007 [16]	Yes	Yes	No	Yes	Yes	No	Yes	5
Hudes, 2007 [17]	Yes	Yes	No	Yes	Yes	No	Yes	5
Jonasch, 2010 [18]	Yes	Yes	No	No	No	No	Yes	3
Procopio, 2011 [19]	Yes	Yes	No	Yes	Yes	No	Yes	5
Rini, 2012 [20]	Yes	Yes	No	No	No	No	Yes	3
Ueda, 2013 [21]	Yes	Yes	No	No	No	No	Yes	3
Motzer, 2013 [22]	Yes	Yes	No	Yes	Yes	No	Yes	5
Rini, 2011 [23]	Yes	Yes	No	Yes	Yes	No	Yes	5
NCT00474786 [24]	Yes	Yes	No	Yes	Yes	No	Yes	5
Celler, 2013 [25]	Yes	Yes	No	Yes	Yes	No	Yes	5
NCT01147822 [26]	Yes	Yes	No	Yes	Yes	No	Yes	5
Bracarda, 2007 [27]	Yes	Yes	No	Yes	Yes	No	Yes	5
Bukowski, 2007[28]	Yes	Yes	No	Yes	Yes	No	Yes	5
NCT00631371 [29]	Yes	Yes	No	Yes	Yes	No	Yes	5
Négrier, 2011 [30]	Yes	Yes	No	Yes	Yes	No	Yes	5

### VEGF(r)-TKI & mTOR inhibitor vs placebo

**Figure 2:**
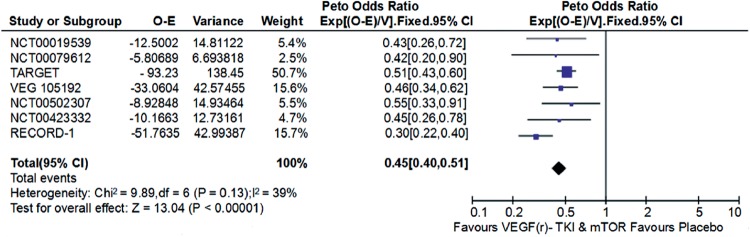
Forest plot and meta-analysis of PFS comparing VEGF(r)-TKI & mTOR inhibitor vs placebo.

**Figure 3:**
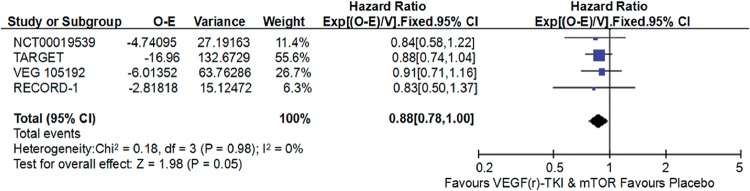
Forest plot and meta-analysis of OS comparing VEGF(r)-TKI & mTOR inhibitor vs placebo.

**Figure 4:**
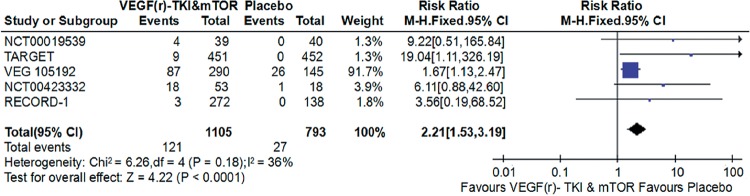
Forest plot and meta-analysis of ORR comparing VEGF(r)-TKI & mTOR inhibitor vs placebo.

Compared with placebo, VEGF(r)-TKI & mTOR inhibitor were associated with improved PFS (HR: 0.45; 95% CI: 0.40-0.51; P<0.001; Figure-2), improved OS (HR: 0.88; 95% CI, 0.78-1.00; P=0.05; Figure-3) and higher ORR (RR: 2.21; 95% CI, 1.53-3.91; P<0.001; Figure-4), respectively.

### VEGF(r)-TKI & mTOR inhibitor vs IFN-α

**Figure 5:**
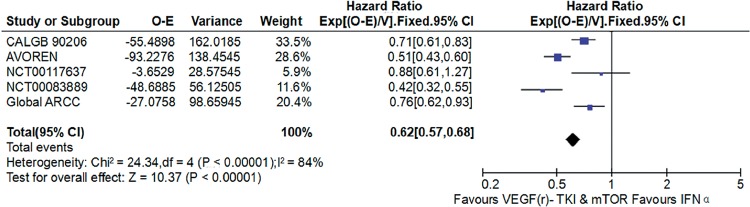
Forest plot and meta-analysis of PFS comparing VEGF(r)-TKI & mTOR inhibitor vs IFN-α.

**Figure 6:**
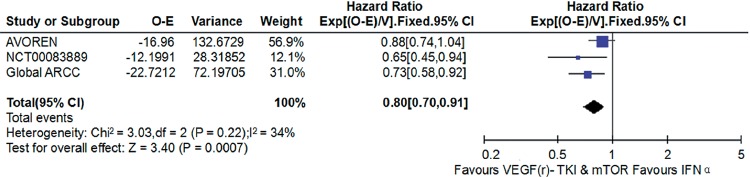
Forest plot and meta-analysis of OS comparing VEGF(r)-TKI & mTOR inhibitor vs IFN-α.

**Figure 7:**
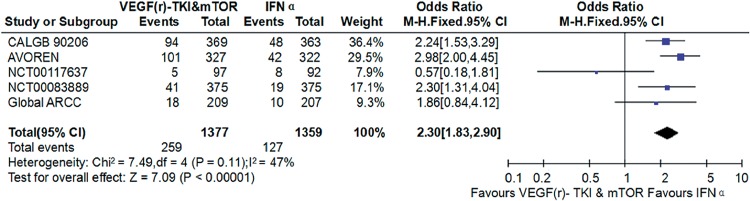
Forest plot and meta-analysis of ORR comparing VEGF(r)-TKI & mTOR inhibitor vs IFN-α.

Compared with IFN-α, VEGF(r)-TKI & mTOR inhibitor were associated with improved PFS (HR: 0.62; 95% CI, 0.57-0.68; P<0.001; Figure-5), improved OS (HR: 0.80; 95% CI, 0.70-0.91; P<0.001; Figure-6) and higher ORR (RR: 2.30; 95% CI, 1.83-2.90; P<0.001; Figure-7), respectively.

### Efficacy of sorafenib and BEV + IFN-α

Three trials (33-35) compared sorafenib combination vs sorafenib; there was no significant difference with regard to PFS (HR: 0.81; 95% CI, 0.59-1.11; P=0.19) and OS (HR: 1.95; 95% CI, 0.84-4.52; P=0.12), but with a higher ORR (RR: 1.51; 95% CI, 1.03-2.22; P=0.03). Three trials (29, 44, 45) compared single or combination VEGF(r)--TKI & mTOR inhibitor vs BEV + IFN-α; there was no significant difference with regard to PFS (HR: 1.08; 95% CI, 0.93-1.25; P=0.31), OS (HR: 1.0; 95% CI, 0.9-1.3; P=0.6), or ORR (RR: 0.85; 95% CI, 0.65-1.12; P=0.26).

### Indirect treatment comparison and the network diagram of HR for PFS

**Figure 8:**
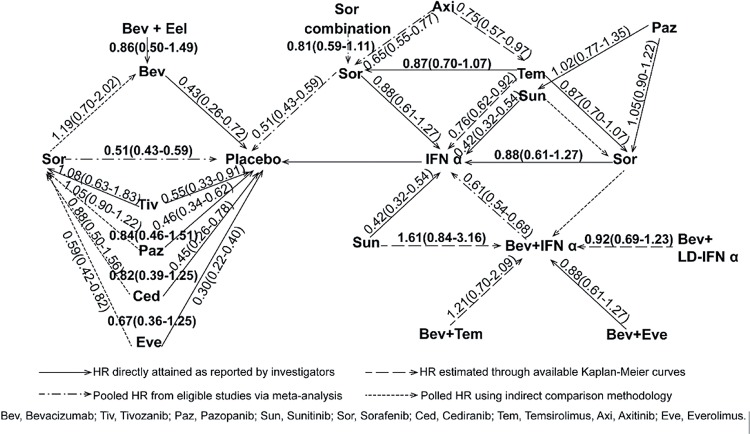
Network diagram of HR for PFS in the current treatments for mRCC.

**Figure 9:**
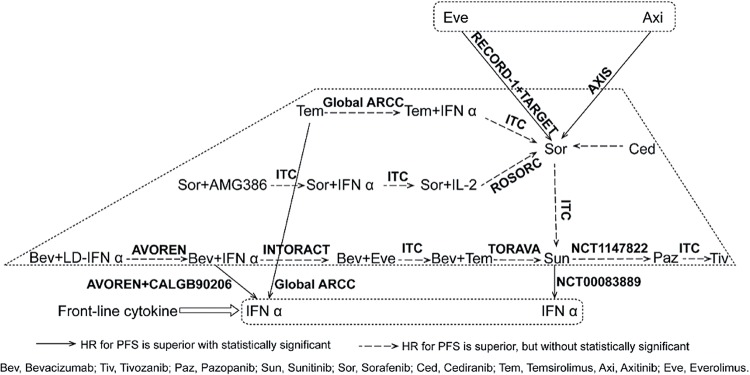
Network diagram of a ranking of the current treatments in order of superiority of HR for PFS.

Pooled HRs by the indirect treatment comparison (ITC) of PFS are listed in Figure-8. By the ITC, axitinib was superior to sorafenib (HR: 0.65; 95% CI, 0.55-0.77) and temsirolimus (HR: 0.75; 95% CI, 0.57-0.97). Everolimus improved PFS versus sorafenib (HR: 0.59; 95% CI, 0.42-0.82). There were no significant differences between the second line targeted therapies as shown in Figure-8. In order of superiority of HR for PFS we made a network diagram of a ranking of the current treatments (Figure-9). Three level ranking system were introduced, that is, IFN-α as the front line cytokine therapy, everolimus and axitinib as the second line agents after failure of initial VEGF(r)-TKI & mTOR inhibitor treatment, and the others remaining as the first line targeted therapies.

## DISCUSSION

Interferon alpha (IFN-α) as the standard of care for mRCC prior to 2005 demonstrated to be associated with limited efficacy and high toxicity in our meta-analysis and only remains an option for front-line cytokine therapy in a small minority of highly selected patients with a good prognosis, which was consistent with the previous studies (47, 48). Treatment for advanced RCC has dramatically changed since 2006 with several targeted agents currently approved for the treatment of advanced RCC, including sunitinib, sorafenib, temsirolimus, everolimus, bevacizumab (in combination with IFN) and pazopanib. With new agents in development, the treatment options for advanced RCC are set to increase further. While head-to-head trials remain the gold standard, as trials need several years to complete, the dynamic advanced RCC treatment environment means that the comparator while appropriate at the time of trial design may not be optimal. In the absence of head-to-head data available at the time of this research, indirect comparisons via systematic review and network meta-analysis provide a robust clinical reference in the evolving treatment of advanced RCC.

### Improved efficacy of VEGF(r)-TKI & mTOR inhibitor vs placebo and IFN-α

Improvements in PFS, OS and ORR benefits vs IFN-α or placebo comparators were demonstrated for all of the available targeted therapies, although some class distinctions were evident between the VEGF(r)-TKI and the mTOR inhibitors. Consistent with their mechanism of action, temsirolimus and everolimus, when used as monotherapy, are primarily cytostatic and appear to affect PFS by stabilizing the disease. Thus, for mTOR inhibitors, although ORR achieved in some patients, might not be clinical benefit in RCC. In the RECORD-1 placebo controlled study, everolimus gained an ORR of 1%, and Global ARCC trial indicated 8.6% versus IFN-α. By contrast, the higher ORRs with sunitinib (11%), sorfenib (2-5.2%), pazopanib (30%), cediranib (34%) or bevacizumab + IFN-α (25.5-31%) obtained in previously untreated patients suggest that tumor regression might play a larger role in the improvement in PFS shown with angiogenesis inhibitors. Eisen et al. also conducted a subgroup analysis of the pivotal TARGET trial which demonstrated that sorafenib conferred a statistically significant increase in PFS and increased clinical benefit. The maintained efficacy combined with an acceptable toxicity profile in both younger and older patients, supports the use of sorafenib as a treatment for advanced RCC in all age groups (49). TIVO-1 (23) trial suggested tivozanib as an orally bioavailable VEGF(r)-TKI had a long half-life and excellent potency and specificity to the VEGF receptors. The drug has shown tolerability and efficacy in early phase trials and has shown superiority to sorafenib in terms of improved PFS and acceptable toxicity profile in patients with metastatic RCC. FDA has not approved tivozanib (50), the activity and safety of tivozanib still required the observation in the ongoing phase III evaluation of tivozanib in patients with advanced or metastatic clear-cell RCC.

### First line targeted therapies of mRCC

Bevacizumab, which has proven to be well tolerated and efficacious in mRCC when combined with cytotoxic chemotherapy, has demonstrated significant clinical benefits in patients with mRCC when combined with IFN-α (51). In the past, there was a consensus that single VEGF(r)-TKI & mTOR inhibitor agents and BEV+IFN are equally effective in terms of PFS in first-line mRCC therapy (52); however, recent publications (53, 54) raised doubts about this comparable efficacy. Our study with expanded-access-study applied indicated a comparable result which is in line with Mickisch et al. findings. Still, number of studies (43-45) explored the combination of bevacizumab with other targeted agents which may display improved efficacy through blockade of the angiogenic pathways at multiple points. However, a recent study in patients with metastatic RCC also showed that the combination of sunitinib plus bevacizumab is not feasible because of a high side reaction of hypertension and vascular and hematological toxicities with chronic therapy (48). Notably, combining bevacizumab with mTOR inhibitors such as everolimus (44) and temsirolimus (45) also out of interest and appears not promising based on preliminary data with attenuated efficacy (HR, 1.1; PFS, 9.1 vs 9.3 mon; ORR, 27 vs 27.4%) and (HR, 1.21; PFS, 8.2 vs 16.8 mon; ORR, 27 vs 43%). And the toxicity profile of the combination of mTOR inhibitors and bevacizumab at full doses of each drug was much higher than anticipated and limited treatment continuation over time. This combination has failed to show any beneficial activity when used as first-li-ne treatment in patients with mRCC and cannot be suitable for recommendation. In conclusion, it is conceivable that bevacizumab monotherapy could provide a safety advantage over its combination with IFN-a, VEGF(r)-TKI and mTOR inhibitor combined chemotherapy.

Sorafenib has been the best-evidenced second-line option after cytokine failure, until the AXIS study discussed below. Subsequent emphasis has been on attempted enhancement of activity by combining sorafenib with other agents, including low dose IFN-α (33), IL-2 (34), and AMG 386 (35). However, no clinically useful advance comparing these combinations with sorafenib alone has been identified. Our network analysis also provides a ranking of the single VEGF(r)-TKI agent treatments in order of superiority. In all trials, axitinib was ranked most likely to be ‘best’, followed by cediranib, sorafenib, sunitinib, pazopanib and tivozanib. Different with the results by James et al. which showed sorafenib followed by pazopanib in superiority according to an indirect comparison with two placebo control trials, we draw the conclusion from the COMPARZ head-to-head studies (30). Also Mills et al. (53) conducted an indirect comparison with IFN-α as the common comparator, finding that sunitinib has a superior potency compared with sorafenib (HR: 0.58, 95% CI, 0.38-0.86) and is associated with a high rate of CR (55, 56). However, in our meta-analysis assessing the efficacy of VEGF(r)-TKI & mTOR inhibitor vs IFN-α, except for the study (30) comparing sorafenib vs IFN-α (HR: 0.88, 95% CI, 0.61-1.27) all remaining studies showed a significant difference, and the one study removed test showed a significant heterogeneity. Considering the inherent limitations, the two IFN-α controlled trials were not suitable for ITC, and finally we performed ITC with two pazopanib controlled trials (25, 26) which may be much credible. Based on the current clinical evidence, BEV+ IFN-α, sorafenib and sunitinib considered as the first-line treatments for metastatic RCC are widely used in patients who have failed prior front cytokine therapy, except in patients with poor-risk features, for whom temsirolimus is the recommended first-line treatment.

Also, two recent trials (29, 42) trying to compare lower versus standard IFN-α combined with bevacizumab and sorafenib, both of which demonstrated that plus frequent low-dose IFN-α enhanced efficacy and tolerability in comparison with standard-dose IFN-α. Alternatively, frequent lower IFN-α may still play a role and warranted to be identified in combination with other available VEGF(r)-TKI agents for the treatment of mRCC.

### Second line targeted therapies of mRCC

Considering the studies identified in the current meta-analysis, both the AXIS (36-38) and RECORD-1 (25) studies enrolled patients or a subgroup of patients who were pretreated with TKIs. However, there were several issues which precluded an appropriate comparison of the relative efficacy of axitinib and everolimus in the TKI-refractory population. For example, patients enrolled in the AXIS study (37) were strictly second line (cytokine & sunitinib-refractory) compared with those enrolled in the RECORD-1 study (38) where all patients had received a minimum of one line of treatment (prior treated with sunitinib & sorafenib) and 79% had received two or more prior treatments. As it would be expected, the analysis findings are consistent with those from the AXIS head-to-head clinical trial (36-38) which indicate that treatment with axitinib has a statistically and clinically significant advantage over treatment with single VEGF(r)-TKI agent alone in terms of PFS in patients with previously treated mRCC in the overall population. Moreover, time to deterioration also favored axitinib, supporting the idea that prolonging disease control connotes clinical benefit in this treatment setting. The tolerability of axitinib generally was similar to sorafenib and other similar VEGF(r)-TKIs. In addition, results by Ueda et al. demonstrated the PFS advantage of axitinib over sorafenib was maintained in Japanese subgroup (36) when time to symptom deterioration was included with the overall efficacy assessment, consistent with the overall population (37, 38) and indicated that axitinib provides extended symptom and disease control for these patients. Furthermore, median PFS and ORR achieved in axitinib treated Japanese patients were longer and higher than those achieved in the overall population treated with axitinib.

Everolimus is the first oral mTOR inhibitor to be evaluated in RCC, and has a different active form from temsirolimus. RECORD-1 (25) compared everolimus with placebo with progressive disease of initial sunitinib and/or sorafenib treatment. The primary endpoint of PFS by independent central review was improved (median PFS 4.9 vs 1.9 months, HR: 0.33, P<0.001). OS was the same in both arms, although everolimus was used in 76% of placebo-assigned patients after disease progression. Our network ITC meta-analysis also indicated a superior PFS (HR: 0.59; 0.42-0.82) compared to sorafenib. Findings here are consistent with a recently published systematic review, which included an adjusted comparison of the effects of treatment with axitinib that was superior compared with sorafenib and pazopanib on PFS for mRCC in terms of PFS (57).

Results from our present study indicated that axitinib and everolimus will be important treatment options to extend PFS that should be considered as effective second-line treatment option in the management of advanced RCC. It is not apparent from comparison of PFS of axitinib in this trial and of everolimus in the RECORD-1 trial that switching mechanism of action or maintaining VEGF suppression is a superior strategy in patients with renal cell carcinoma. Further information to determine the optimal treatment algorithm in the second-line management of advanced RCC with regard to the sequence of treatments may come from ongoing trials (58). However, it is important that further robust head-to-head RCTs must be carried out in order to assess the relative efficacies of treatments in a clinically relevant population, that is, after failure of initial VEGF-targeted therapy. In conclusion, the present systematic review/meta-analysis indicated that recently raising targeted agents, axitinib and everolimus as the second-line setting, may offer improvements in terms of PFS compared with the more established agents.

### Evidence strengths and limitations

**Supplementary Figure 2:**
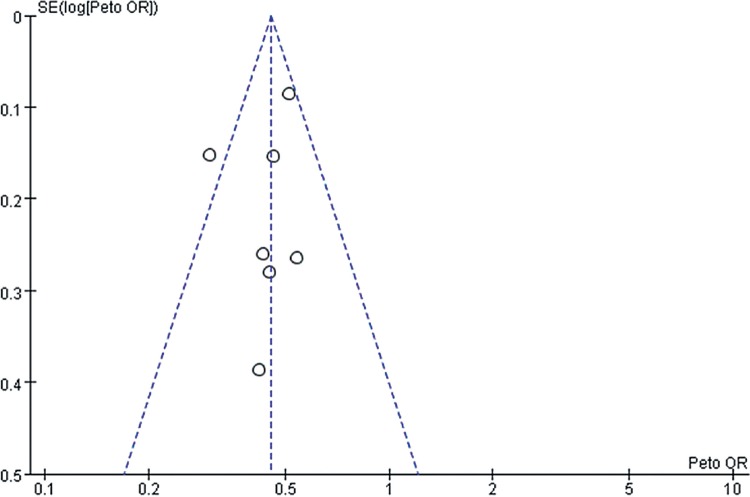
The funnel plot for meta-analysis of PFS comparing VEGF(r)-TKI & mTOR inhibitor vs placebo.

**Supplementary Figure 3:**
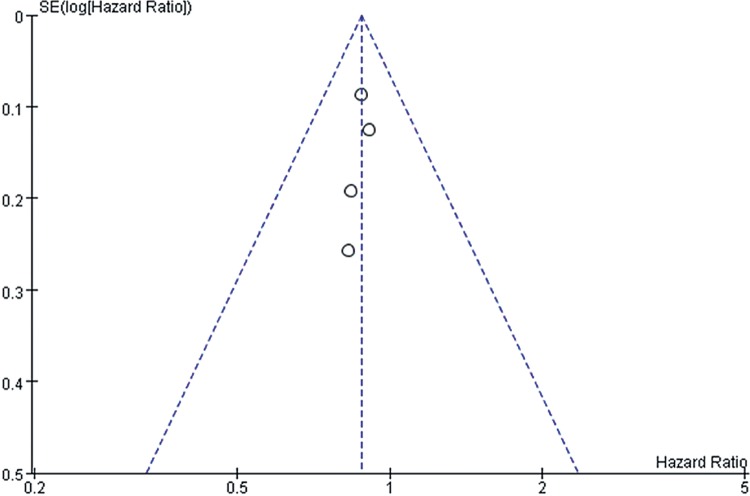
The funnel plot for meta-analysis of OS comparing VEGF(r)-TKI & mTOR inhibitor vs placebo.

**Supplementary Figure 4:**
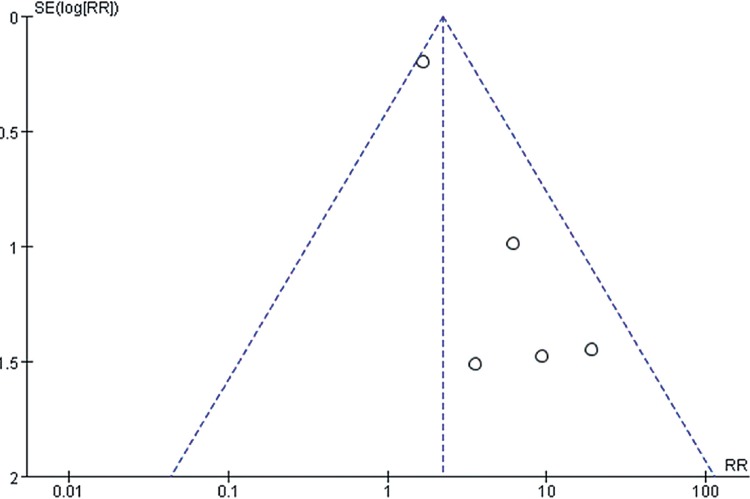
The funnel plot for meta-analysis of ORR comparing VEGF(r)-TKI & mTOR inhibitor vs placebo.

**Supplementary Figure 5:**
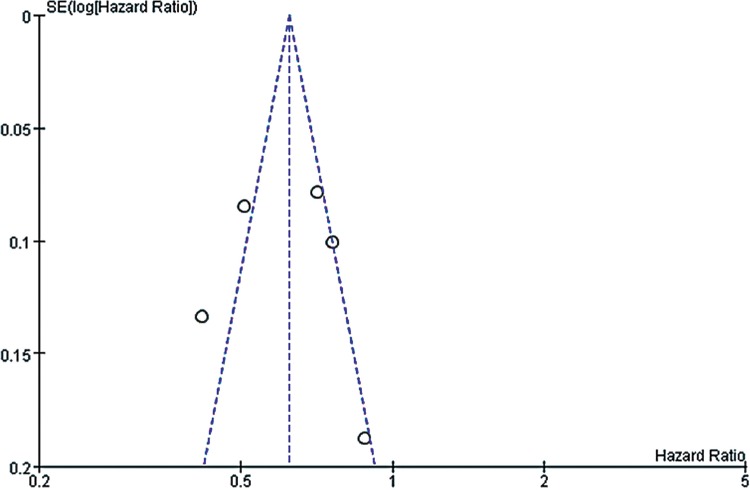
The funnel plot for meta-analysis of PFS comparing VEGF(r)-TKI & mTOR inhibitor vs IFN-α.

**Supplementary Figure 6:**
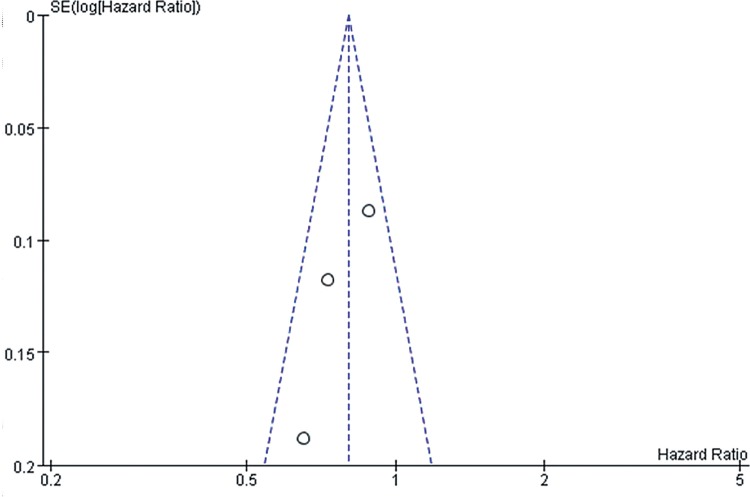
The funnel plot for meta-analysis of OS comparing VEGF(r)-TKI & mTOR inhibitor vs IFN-α.

**Supplementary Figure 7:**
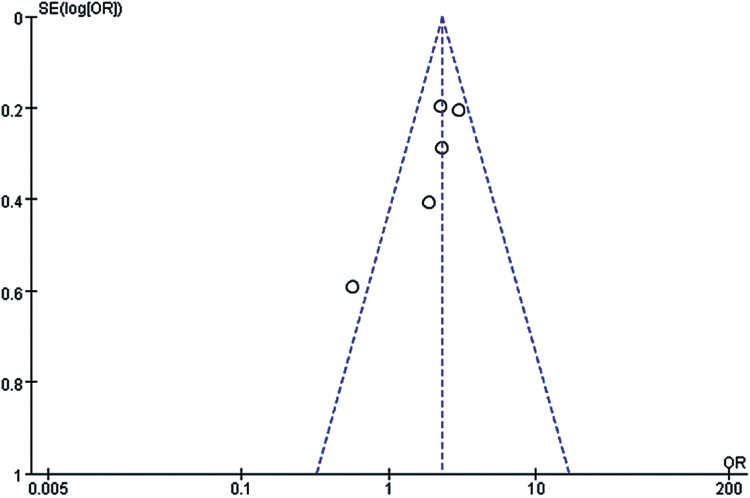
Forest plot and meta-analysis of ORR comparing VEGF(r)-TKI & mTOR inhibitor vs IFN-α.

However, we should admit that there existed certain inherent limitations in the trials included in our meta-analysis that cannot be ignored when interpreting our data. The major limitation is that our findings are partially based on indirect evidence. Although ITC allows indirect estimates to be calculated, they can be subject to potential biases and uncertainties (59). Such an indirect treatment comparison has to be regarded as a complementary assessment to clinical trials, because it cannot substitute direct evidence. However, in the absence of any head-to-head comparison, the indirect treatment comparison approach should be regarded as the most valuable way of estimating treatment effects in a statistically accurate manner (60). A systematic review and meta-analysis was conducted at an appropriate time with enough high quality data available for extraction by a comprehensive and robust search strategy. Also, the statistical power of this systematic review was limited by the small sample sizes of these studies, which ranged from 54 to 903 participants. It is well known that smaller studies are prone to publication bias and generate less reliable estimates of the size effect for any association. In Supplementary Figures 2-7, these funnel plots show an asymmetrical distribution of studies with low statistical power clearly. We applied a rigorous inclusion/exclusion criterion, different subgroups to identify studies, fully outcomes of interest (PFS, OS, ORR), bias adjusted data, strict criteria with Jadad scales to evaluate the quality of the included studies, and advanced network analysis of HR for PFS. Here, we provide up-to-date information of the network diagram of HR for PFS with regard to the current targeted therapies on mRCC which may worth reference on the clinical decision.

In conclusion, our data suggest that targeted therapy with VEGF(r)-TKI & mTOR inhibitor is associated with superior efficacy for treating advanced RCC with improved PFS, OS and higher ORR compared to placebo and IFN-α. Agents targeting VEGF and mTOR pathways improve PFS in both first-line and second-line settings. In the light of this available evidence, there is no statistically significant PFS difference between BEV+IFN and TKIs in first-line mRCC therapy. Network diagram of pooled HR for PFS demonstrates axitinib and everolimus were more effective as the second line agents after failure of an initial VEGF(r)-TKI & mTOR treatment. In summary, here we give a comprehensive overview of current targeted therapies of advanced RCC and it may provide an evidence for the adequate targeted therapy selection. While acknowledging inherent bias in indirect treatment comparisons, upon consideration of each of the factors outlined in this review, the adequate treatment decision criteria of mRCC with targeted therapies remain considered with the safety and tolerability of agents and further robust large sample head-to-head RCTs are warranted to confirm our conclusion.

## APPENDIX

**SUPPLEMENTARY PROTOCOL**

**Indirect treatment comparison (ITC) of everolimus vs. sorafenib for PFS**

**1. Overview of selected RCTs reported everolimus and sorafenib**

**Table:**

Reference	Trial name	Intervention	Comparator	Patients	Outcomes	PFS	HR	P
Ratain, 2006	NCT00079612	Sorafenib	Placebo	32/33	**PFS,** ORR	5.5/1.4	0.42 (0.20-0.91)	0.009
Escudier, 2007	TARGET	Sorafenib	Placebo	451/452	OS, **PFS,** ORR	5.5/2.8	0.51 (0.43-0.60)	<0.0001
Motzer, 2008	RECORD-1	Everolimus	Placebo	272/138	OS, **PFS**, ORR	4.0/1.9	0.30 (0.22-0.40)	<0.0001

**2. Pooled HR for PFS comparing sorafenib versus placebo via meta-analysis**

**3. Indirect treatment comparison: efficacy connections between the pivotal trials**

**4. Indirect comparison methodological procedures in detail**

**Table:**

	Description	Formula	Everolimus	Sorafenib
Start	Basis data: HR for PFS vs PBO	PFS HR (95% CI) vs PBO	0.30 (0.22-0.40)	0.51 (0.43-0.59)
Step 1	Calculation of the Ln (HR)	Ln (HR)	-1.20	-0.67
Step 2	Calculation of the HR_ITC_	HR_ITC_ = EXP{Ln(HR)_Eve_-LN(HR)_Sor_}	0.59	
Step 3	Calculation of the SE Ln (HR)	SE_Ln(HR)_ = {Ln(UCI)-Ln(LCI)}/3.92	0.15	0.08
Step 4	Calculation of the SE_ITC_	SE_ITC_ = SQRT(SE_Eve_^2^ + SE_Sor_^2^)	0.17	
Step 5	Calculation of the 95% CI_ITC_	95% CI_ITC_ = EXP{Ln(HR_ITC_)±1.96×SE_ITC_}	(0.42-0.82)	
Result	ITC HR (Everolimus vs Sorfenib) = 0.59 (95% CI: 0.42-0.82)			

**Eve =** Everolimus; **Sor =** Sorafenib; **PBO =** Placebo; **ITC =** indirect treatment comparison; **PFS =** progression free survival; **HR =** hazard ratio; **95% CI =** 95% confidence interval; **SE =** standard error; **UCI =** upper CI; **LCI =** lower CI.

**5. Network diagram of a ranking of the three agents in order of superiority of HR**

## References

[1] Cohen HT, McGovern FJ (2005). Renal-cell carcinoma. N Engl J Med.

[2] Vogelzang NJ, Stadler WM (1998). Kidney cancer. Lancet.

[3] Coppin C, Porzsolt F, Awa A, Kumpf J, Coldman A, Wilt T (2005). Immunotherapy for advanced renal cell cancer. Cochrane Database Syst Rev.

[4] Nese N, Paner GP, Mallin K, Ritchey J, Stewart A, Amin MB (2009). Renal cell carcinoma: assessment of key pathologic prognostic parameters and patient characteristics in 47,909 cases using the National Cancer Data Base. Ann Diagn Pathol.

[5] Garcia JA, Rini BI (2007). Recent progress in the management of advanced renal cell carcinoma. CA Cancer J Clin.

[6] Mickisch GH (2003). Rational selection of a control arm for randomised trials in metastatic renal cell carcinoma. Eur Urol.

[7] Molina AM, Motzer RJ (2011). Clinical practice guidelines for the treatment of metastatic renal cell carcinoma: today and tomorrow. Oncologist.

[8] Mulders P (2009). Vascular endothelial growth factor and mTOR pathways in renal cell carcinoma: differences and synergies of two targeted mechanisms. BJU Int.

[9] Mills EJ, Bansback N, Ghement I, Thorlund K, Kelly S, Puhan MA (2011). Multiple treatment comparison meta-analyses: a step forward into complexity. Clin Epidemiol.

[10] Jadad AR, Moore RA, Carroll D, Jenkinson C, Reynolds DJ, Gavaghan DJ (1996). Assessing the quality of reports of randomized clinical trials: is blinding necessary?. Control Clin Trials.

[11] Parmar MK, Torri V, Stewart L (1998). Extracting summary statistics to perform meta-analyses of the published literature for survival endpoints. Stat Med.

[12] Tierney JF, Stewart LA, Ghersi D, Burdett S, Sydes MR (2007). Practical methods for incorporating summary time-to-event data into meta-analysis. Trials.

[13] Bucher HC, Guyatt GH, Griffith LE, Walter SD (1997). The results of direct and indirect treatment comparisons in metaanalysis of randomized controlled trials. J Clin Epidemiol.

[14] Woods BS, Hawkins N, Scott DA (2010). Network meta-analysis on the log-hazard scale, combining count and hazard ratio statistics accounting for multi-arm trials: a tutorial. BMC Med Res Methodol.

[15] Wells GA, Sultan SA, Chen L, Khan M, Coyle D, CADTH, Canada (2009). Indirect evidence indirect treatment comparisons in meta-analysis. CADTH Technology Report.

[16] Yang JC, Haworth L, Sherry RM, Hwu P, Schwartzentruber DJ, Topalian SL (2003). A randomized trial of bevacizumab, na anti-vascular endothelial growth factor antibody, for metastatic renal cancer. N Engl J Med.

[17] Ratain MJ, Eisen T, Stadler WM, Flaherty KT, Kaye SB, Rosner GL (2006). Phase II placebo-controlled randomized discontinuation trial of sorafenib in patients with metastatic renal cell carcinoma. J Clin Oncol.

[18] Escudier B, Eisen T, Stadler WM, Szczylik C, Oudard S, Siebels M, TARGET Study Group (2007). Sorafenib in advanced clear-cell renal-cell carcinoma. N Engl J Med.

[19] Escudier B, Eisen T, Stadler WM, Szczylik C, Oudard S, Staehler M (2009). Sorafenib for treatment of renal cell carcinoma: Final efficacy and safety results of the phase III treatment approaches in renal cancer global evaluation trial. J Clin Oncol.

[20] Borregaard J, Ersbøll J, ten Bosch GJ, van Zwieten-Boot B, Abadie E (2011). The European Medicines Agency review of pazopanib for the treatment of advanced renal cell carcinoma: summary of the scientific assessment of the Committee for Medicinal Products for Human Use. Clin Cancer Res.

[21] Sternberg CN, Davis ID, Mardiak J, Szczylik C, Lee E, Wagstaff J (2010). Pazopanib in locally advanced or metastatic renal cell carcinoma: results of a randomized phase III trial. J Clin Oncol.

[22] Sternberg CN, Hawkins RE, Wagstaff J, Salman P, Mardiak J, Barrios CH (2013). A randomised, double-blind phase III study of pazopanib in patients with advanced and/or metastatic renal cell carcinoma: final overall survival results and safety update. Eur J Cancer.

[23] Nosov DA, Esteves B, Lipatov ON, Lyulko AA, Anischenko AA, Chacko RT (2012). Antitumor activity and safety of tivozanib (AV-951) in a phase II randomized discontinuation trial in patients with renal cell carcinoma. J Clin Oncol.

[24] Mulders P, Hawkins R, Nathan P, de Jong I, Osanto S, Porfiri E (2012). Cediranib monotherapy in patients with advanced renal cell carcinoma: results of a randomised phase II study. Eur J Cancer.

[25] Motzer RJ, Escudier B, Oudard S, Hutson TE, Porta C, Bracarda S, RECORD-1 Study Group (2008). Efficacy of everolimus in advanced renal cell carcinoma: a doubleblind, randomised, placebo-controlled phase III trial. Lancet.

[26] Rini BI, Halabi S, Rosenberg JE, Stadler WM, Vaena DA, Ou SS (2008). Bevacizumab plus interferon alfa compared with interferon alfa monotherapy in patients with metastatic renal cell carcinoma: CALGB 90206. J Clin Oncol.

[27] Melichar B, Bracarda S, Matveev V, Alekseev B, Ivanov S, Zyryanov A (2013). A multinational phase II trial of bevacizumab with low-dose interferon-α2a as first-line treatment of metastatic renal cell carcinoma: BEVLiN. Ann Oncol.

[28] Escudier B, Pluzanska A, Koralewski P, Ravaud A, Bracarda S, Szczylik C (2007). Bevacizumab plus interferon alfa-2a for treatment of metastatic renal cell carcinoma: a randomised, double-blind phase III trial. Lancet.

[29] Escudier B, Bellmunt J, Négrier S, Bajetta E, Melichar B, Bracarda S (2010). Phase III trial of bevacizumab plus interferon alfa-2a in patients with metastatic renal cell carcinoma (AVOREN): final analysis of overall survival. J Clin Oncol.

[30] Escudier B, Szczylik C, Hutson TE, Demkow T, Staehler M, Rolland F (2009). Randomized phase II trial of firstline treatment with sorafenib versus interferon Alfa-2a in patients with metastatic renal cell carcinoma. J Clin Oncol.

[31] Motzer RJ, Hutson TE, Tomczak P, Michaelson MD, Bukowski RM, Rixe O (2007). Sunitinib versus interferon alfa in metastatic renal-cell carcinoma. N Engl J Med.

[32] Hudes G, Carducci M, Tomczak P, Dutcher J, Figlin R, Kapoor A (2007). Temsirolimus, interferon alfa, or both for advanced renal-cell carcinoma. N Engl J Med.

[33] Jonasch E, Corn P, Pagliaro LC, Warneke CL, Johnson MM, Tamboli P (2010). Upfront, randomized, phase 2 trial of sorafenib versus sorafenib and low-dose interferon alfa in patients with advanced renal cell carcinoma: clinical and biomarker analysis. Cancer.

[34] Procopio G, Verzoni E, Bracarda S, Ricci S, Sacco C, Ridolfi L (2011). Sorafenib with interleukin-2 vs sorafenib alone in metastatic renal cell carcinoma: the ROSORC trial. Br J Cancer.

[35] Rini B, Szczylik C, Tannir NM, Koralewski P, Tomczak P, Deptala A (2012). AMG 386 in combination with sorafenib in patients with metastatic clear cell carcinoma of the kidney: a randomized, double-blind, placebo-controlled, phase 2 study. Cancer.

[36] Ueda T, Uemura H, Tomita Y, Tsukamoto T, Kanayama H, Shinohara N (2013). Efficacy and safety of axitinib versus sorafenib in metastatic renal cell carcinoma: subgroup analysis of Japanese patients from the global randomized Phase 3 AXIS trial. Jpn J Clin Oncol.

[37] Motzer RJ, Escudier B, Tomczak P, Hutson TE, Michaelson MD, Negrier S (2013). Axitinib versus sorafenib as second-line treatment for advanced renal cell carcinoma: overall survival analysis and updated results from a randomised phase 3 trial. Lancet Oncol.

[38] Rini BI, Escudier B, Tomczak P, Kaprin A, Szczylik C, Hutson TE (2011). Comparative effectiveness of axitinib versus sorafenib in advanced renal cell carcinoma (AXIS): a randomised phase 3 trial. Lancet.

[39] Xiao W, Wang J, Li H, Guan W, Xia D, Yu G (2013). Fibulin-1 is down-regulated through promoter hypermethylation and suppresses renal cell carcinoma progression. J Urol.

[40] Motzer RJ, Nosov D, Eisen T, Bondarenko I, Lesovoy V, Lipatov O (2013). Tivozanib versus sorafenib as initial targeted therapy for patients with metastatic renal cell carcinoma: results from a phase III trial. J Clin Oncol.

[41] Yu G, Yao W, Wang J, Ma X, Xiao W, Li H (2012). LncRNAs expression. signatures of renal clear cell carcinoma revealed by microarray. PLoS One.

[42] Bracarda S, Porta C, Boni C, Santoro A, Mucciarini C, Pazzola A (2013). Could interferon still play a role in metastatic renal cell carcinoma? A randomized study of two schedules of sorafenib plus interferon-alpha 2a (RAPSODY). Eur Urol.

[43] Bukowski RM, Kabbinavar FF, Figlin RA, Flaherty K, Srinivas S, Vaishampayan U (2007). Randomized phase II study of erlotinib combined with bevacizumab compared with bevacizumab alone in metastatic renal cell cancer. J Clin Oncol.

[44] Wheler JJ, Moulder SL, Naing A, Janku F, Piha-Paul SA, Falchook GS (2014). Anastrozole and everolimus in advanced gynecologic and breast malignancies: activity and molecular alterations in the PI3K/AKT/mTOR pathway. Oncotarget.

[45] Négrier S, Gravis G, Pérol D, Chevreau C, Delva R, Bay JO (2011). Temsirolimus and bevacizumab, or sunitinib, or interferon alfa and bevacizumab for patients with advanced renal cell carcinoma (TORAVA): a randomised phase 2 trial. Lancet Oncol.

[46] Negrier S, Escudier B, Lasset C, Douillard JY, Savary J, Chevreau C (1998). Recombinant human interleukin-2, recombinant human interferon alfa-2a, or both in metastatic renal-cell carcinoma. Groupe Français d'Immunothérapie. N Engl J Med.

[47] Negrier S, Escudier B, Lasset C, Douillard JY, Savary J, Chevreau C (1998). Recombinant human interleukin-2, recombinant human interferon alfa-2a, or both in metastatic renal-cell carcinoma. Groupe Français d'Immunothérapie. N Engl J Med.

[48] Atkins MB, Regan M, McDermott D (2004). Update on the role of interleukin 2 and other cytokines in the treatment of patients with stage IV renal carcinoma. Clin Cancer Res.

[49] Eisen T, Oudard S, Szczylik C, Gravis G, Heinzer H, Middleton R (2008). Sorafenib for older patients with renal cell carcinoma: subset analysis from a randomized trial. J Natl Cancer Inst.

[50] Cowey CL (2013). Profile of tivozanib and its potential for the treatment of advanced renal cell carcinoma. Drug Des Devel Ther.

[51] McDermott DF, George DJ (2010). Bevacizumab as a treatment option in advanced renal cell carcinoma: an analysis and interpretation of clinical trial data. Cancer Treat Rev.

[52] Coppin C, Le L, Porzsolt F, Wilt T (2008). Targeted therapy for advanced renal cell carcinoma. Cochrane Database Syst Rev.

[53] Mills EJ, Rachlis B, O'Regan C, Thabane L, Perri D (2009). Metastatic renal cell cancer treatments: an indirect comparison metaanalysis. BMC Cancer.

[54] Thompson Coon JS, Liu Z, Hoyle M, Rogers G, Green C, Moxham T (2009). Sunitinib and bevacizumab for first-line treatment of metastatic renal cell carcinoma: a systematic review and indirect comparison of clinical effectiveness. Br J Cancer.

[55] Albiges L, Oudard S, Negrier S, Caty A, Gravis G, Joly F (2012). Complete remission with tyrosine kinase inhibitors in renal cell carcinoma. J Clin Oncol.

[56] Wood L (2012). Sunitinib malate for the treatment of renal cell carcinoma. Expert Opin Pharmacother.

[57] Larkin J, Paine A, Tumur I, Cappelleri JC, Healey PJ, Foley G (2013). Second-line treatments for the management of advanced renal cell carcinoma: systematic review and metaanalysis. Expert Opin Pharmacother.

[58] Motzer RJ, Barrios CH, Kim TM, Falcon S, Cosgriff T, Harker WG (2014). Phase II randomized trial comparing sequential first-line everolimus and second-line sunitinib versus first-line sunitinib and second-line everolimus in patients with metastatic renal cell carcinoma. J Clin Oncol.

[59] Glenny AM, Altman DG, Song F, Sakarovitch C, Deeks JJ, D'Amico R (2005). Indirect comparisons of competing interventions. Health Technol Assess.

[60] Mickisch GH, Schwander B, Escudier B, Bellmunt J, Maroto JP, Porta C (2011). Indirect treatment comparison of bevacizumab + interferon-α-2a vs tyrosine kinase inhibitors in first-line metastatic renal cell carcinoma therapy. Clinicoecon Outcomes Res.

